# Supplementary figures and images for: Clinical, Histopathologic, and Immunohistochemical Features of Patients with IgG/IgA Pemphigus
Source: Biomedicines. 2022 May 22;10(5):1197. doi: 10.3390/biomedicines10051197 (PMC9138426; doi:10.3390/biomedicines10051197)

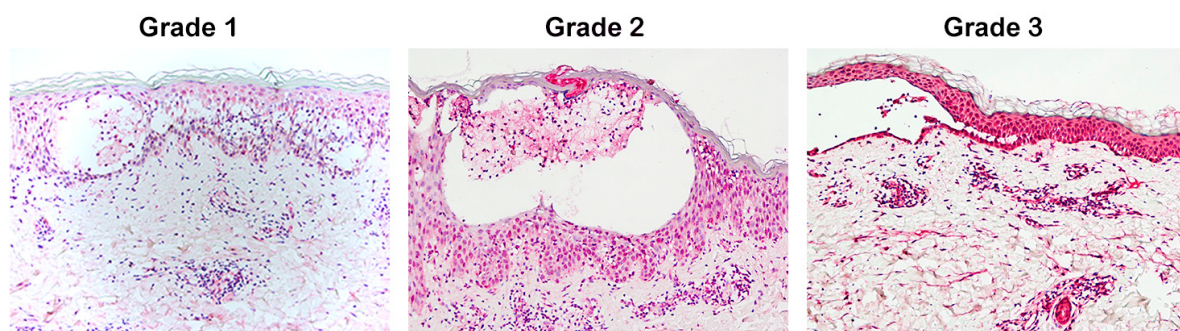

Figure S1: The representative images of IHC grading.

Supplement: Supplementary file 1 [file biomedicines-10-01197-s001.zip › biomedicines-1705866-supplementary.pdf]
